# Effective gene delivery using size dependant nano core-shell in human cervical cancer cell lines by magnetofection

**DOI:** 10.1371/journal.pone.0289731

**Published:** 2023-09-07

**Authors:** Srinivasa Sundara Rajan R., Jobin Thomas, Dileep Francis, Elcey C. Daniel

**Affiliations:** 1 Biotechnology Research Centre, Kristu Jayanti College (Autonomous), Bengaluru, Karnataka, India; 2 Centre for Nano Bbiotechnology (CNBT), Vellore Institute of Technology, Vellore, Tamil Nadu, India; VIT University, INDIA

## Abstract

Biocompatible magnetic nanoparticles are effective for gene delivery in vitro and in vivo transfection. These mediators are mainly used to deliver drugs and genes. It can also be used as probes to diagnose and treat various diseases. Magnetic nanoparticles, primarily iron oxide nanoparticles, are used in various biological applications. However, preparing stable and small-size biocompatible core-shell is crucial in site direct gene delivery. In the present study, superparamagnetic iron oxide nanoparticles were synthesized using the chemical co-precipitation method and were functionalized with starch to attain stable particles. These SPIONs were coated with polyethylenimine to give a net positive charge. The fluorescent plasmid DNA bound to the SPIONs were used as a core shell for gene delivery into the HeLa cells via magnetofection. UV-Visible Spectrophotometry analysis showed a peak at 200 nm, which confirms the presence of FeO nanoparticles. The Scanning Electron Microscopy images revealed the formation of spherical-shaped nanoparticles with an average size of 10 nm. X-ray Diffraction also confirmed FeO as a significant constituent element. Vibrating Sample Magnetometry ensures that the nanoparticles are superparamagnetic. Atomic Force Microscopy images show the DNA bound on the surface of the nanoparticles. The gene delivery and transfection efficiency were analyzed by flow cytometry. These nanoparticles could effectively compact the pDNA, allowing efficient gene transfer into the HeLa cell lines.

## Introduction

The recent development of nonviral transfection agents for gene delivery has given rise to high efficiency. Gene therapy is the latest technique to treat various genetic disorders. Introducing the desired gene, alters defective genes and restores normal metabolism. Magnetic nanoparticles have been used as an efficient carrier in delivering specific therapeutic agents, which are attached or encapsulated within a magnetic nanoparticle. Magnetic nanoparticles composed of an iron oxide core and polymeric shell present an exceptional carrier for gene delivery of nucleic acids. The high magnetic property of the iron oxide core enables the non-invasive administration of magnetic nanoparticles by magnetic fields. The nanoparticle’s surface is responsible for interaction with the drug and host.

Many methods have been developed for synthesizing iron oxide nanoparticles (NPs), where one can control its shape, stability, biocompatible and monodispersed iron oxide NPs. The most common method includes co-precipitation and thermal decomposition. Other methods include hydrothermal synthesis, microemulsion, and sonochemical synthesis [1]. Co-precipitation is the best method for obtaining Fe_3_O_4_ and *γ*-Fe_2_O_3_ [2].

The iron oxide Nanoparticle’s surface has to be functionalized with a suitable stabilizer to prevent agglomeration and to obtain a stable colloidal solution. Surfactants aid in controlling the particle size and stabilizing the colloidal dispersions [1]. Starch, a branched hydrophilic long-chain polymer of D-glucose, is being used as a drug carrier due to its biocompatibility, nontoxicity, and biodegradability [3]. When starch is combined with colloidal particles, the magnetic stability rises to various applications, such as gene delivery, drug delivery, magnetic resonance imaging, and tissue engineering. Due to its neutral free hydroxyl functional groups, it can bind to diverse chemical groups and ions and enhance surface activity [4, 5].

The coating of iron oxide NPs with polymers has recently received more attention. Polymer coating will increase repulsive forces to balance the magnetic and the Van der Waals attractive forces acting on the NPs. Natural polymers like dextran, starch, chitosan, or synthetic polymers like polyethylene glycol (PEG), polymethylmethacrylate (PMMA), and polyethylenimine (PEI) can be utilized, which are biocompatible. Size control, high encapsulation efficiency, and sustained release behaviour of the anticancer drug are shown by biodegradable polymer Nanoparticles [6]. PEI is the most widely used biopolymer agent capable of forming complexes with DNA, condensing them into compact nanoparticles, and protecting DNA against degradation [7]. Based on their high surface charge, PEIs are promising candidates for the delivery of negatively charged nucleic acids. PEI attached to nanoparticle surfaces through covalent and electrostatic interactions achieves the goal [8]. The transfection capability and cytotoxicity effect depend on the molecular weight and structure of PEIs [6]. The “proton sponge effect” is responsible for their high transfection efficiency. This property is thought to lead to buffering inside endosomes. The osmotic swelling and physical rupture of the endosomes are due to the pumping of protons into the endosomes, which helps escape vectors from the degradative lysosomal pathway [9]. A lower concentration of PEI needs to be used to reduce its toxicity for the effective core shell [10].

Magnetofection combines nucleic acid and its vector with magnetic nanoparticles, so they can be drawn and concentrated to the target cells by applying a magnetic field. This technique enhances the efficiency up to several hundred folds and can reduce the process from 4 h to 15 min. This process requires magnetic nanoparticles to be surface functionalized to couple with the gene complexes [7]. Magnetic nanoparticle functionalized with PEI binds to DNA and acts efficiently in in-vitro gene transfer [11].

The present study emphasizes the synthesis of superparamagnetic iron oxide nanoparticles (SPION) using the co-precipitation method. These synthesized nanoparticles were functionalized with potato starch and coated with PEI. Further, the core-shell was prepared by binding the plasmid DNA on the PEI surface. The core shell was used for gene delivery onto cell lines by magnetofection. Gene delivery and transfection efficiency was analyzed using flow cytometry.

## Materials and methods

### Material

For the synthesis of iron oxide nanoparticles, ferrous sulphate heptahydrate (FeSO_4_.7H_2_O) was purchased from HiMedia Laboratories, India. Ferric chloride hexahydrate (FeCl_3_.6H_2_O) was purchased from Thomas Baker (Chemicals) Pvt. Ltd., India. Tetramethyl ammonium hydroxide (C_4_H_13_NO), formaldehyde (CH_2_O), acetone (C_3_H_6_O), Glucose, EDTA, NaOH and Potassium Acetate, Tris Buffer, and starch were purchased from SD Finechem Limited, India. Polyethyleneimine was procured from TCI CO., Ltd. N, N Dimethylformamide was purchased from Fisher Scientific, Pvt., Ltd. For proper solution mixing during the incubation, a cyclo-rotator was designed to fit our purpose. HeLa cells were obtained from Stellixer Biotec, Bengaluru, India.

### Plasmid DNA isolation

The plasmid DNA was isolated from the DH5α strain of *E*.*coli* with pBSK, using the standard method [12]. The isolated plasmid DNA was purified and stored in TE buffer at 4°C.

### Preparation of superparamagnetic iron oxide nanoparticles (SPION)

Preparation of SPIONs were carried out according to the protocol mentioned by Justin et al [13] with slight modifications. Equal quantities (0.1 g) of FeCl_3_.6H_2_O and FeSO_4._7H_2_O were dissolved in 1 ml of nitrogenized double distilled water. 50*μ* l of the above solution was mixed with 500 *μ*l tetramethyl ammonium hydroxide and vortexed to obtain a black-coloured precipitate. The resultant black residue (20 *μ*l) was washed in 1 ml acetone, and with the help of a magnetic field, acetone was slowly pipetted out. The pellet was dried in a hot air oven (60 °C) for half an hour to remove acetone and later dissolved in 800 *μ*l of formaldehyde. Then, 400 *μ*l of nitrogenized double distilled water was added dropwise, followed by 300 *μ*l of formaldehyde solution. This solution was kept in a magnetic field for three days.

### Stabilization and functionalization of SPIONs

For stabilization of SPIONs, 35μg of synthesized SPIONs were washed with water under a magnetic field. Added 5 ml of N, N dimethylformamide, and 10 ml starch solution (0.08%) to the washed SPIONs. Further, the solution was kept in a cyclo-rotator for two days, which rotates once every 30 minutes.

### Polymer coating of SPIONs

Starch functionalized SPIONs (5ml) were added with 10 ml of PEI solution (5%) for the polymer coating and kept in a cyclo-rotator for two days.

### Preparation of SPIONs-DNA (core shell) complexes

The core shell was synthesized by adding isolated plasmid DNA (20 μl of 1μg/μl concentration) to 10 ml of polymer-coated SPIONs. The solution was placed in a cyclo-rotator for two days.

### Analytical methods

In order to analyze the presence of the SPIONs, UV–Visible spectroscopy (UV Vis) analysis of the samples was recorded between 200 and 800 nm in the V-650 Jasco UV-Vis spectrophotometer. The Fourier-transform Infrared spectra (FTIR) were recorded at transmission mode scan for the spectral region between 4000–450 cm^-1^ using Perkin Elmer Spectrum 1 FT-IR for the confirmation of SPIONs. Raman spectrum of the material was recorded using Bruker. The stability of nanoparticles was investigated by Zeta potential analysis performed using Zetasizer Nano ZS. Liquid samples were directly used to analyze UV Vis, FTIR, Raman, and Zeta potential. Samples were drop casted, dried on a 1 cm^2^ slide, fitted onto a grid, and X-ray diffraction (XRD) was recorded using the Rigaku model. The crystalline size can be determined using Scherrer’s equation:

D=kλ/βcosθ

K is Scherrer constant (0.94), λ is X-ray wavelength 1.54, and β is the half-intensity width of the diffraction peak. The reflecting peak at 2*θ* = 45.46 was chosen for calculation.

Scanning Electron Microscope (SEM) images were taken in Zeiss Ultra 55. Before SEM imaging, the samples were loaded onto a silicon grid and gold spurted with Quorum Q 150R ES. Size of the core-shell also confirmed by Dynamic light scattering, performed in Brookhaven instruments. Magnetic measurement of the sample was recorded using Vibrating Sample Magnetometry (VSM) Lakeshore VSM 7410. The magnetic particle size can also be calculated from the hysteresis curve using the following formula:

$$D_{m}={\left((18K_{B}T/\pi )(x_{I}/\rho Ms)\right)}^{1/3}$$

Slope3.56548E-5

Here x_i_ is the initial magnetic susceptibility (*χ*) x_i_ = (dM/dH)_H 0_ cm^3^mol^-1^ and *ρ* is the density of FeO (5.79 g/ cm3) and KB Boltzmann constant. The initial slope was calculated from the hysteresis plots.

Atomic Force Microscope (AFM) images of the samples were captured to compare the materials prepared from SPIONs to the core-shell, using 5500 series AFM, Agilent technologies and were analyzed using WSxM imaging software.

### Agarose gel retardation

The plasmid DNA binding ability of the nanoparticles was determined by agarose gel (1.0% w/v) retardation assay. The core-shell and plasmid DNA were mixed in 1:1, 5:1, 10:1, and 20:1 ratios. The concentration of plasmid DNA was 0.5 *μg*/*μl*^−1^, and the weight of nanoparticles was 0.5 *μ*g. The DNA-coated core shell was subjected to agarose gel electrophoresis to analyze the bound DNA at 100 v, and the gel was analyzed using a UV transilluminator.

### Cytotoxicity studies

Cell suspension (HeLa cell—100μl) was seeded in a 96-well plate at the required cell density (25,000–50,000 cells per well) and allowed the cells to grow for 12 h. Different concentrations of nanoparticle core-shell were added to the culture well and transfected in the presence and absence of a magnetic field. The total volume in the well was made into 200 μl using media. The plates were incubated at 37 °C for 24 hrs in a 5% CO_2_ incubator. Media in the plates were drained after observing the growth of the cells and added with 10µl of MTT. The cells were incubated for two hours in a dark place. After incubation, the MTT reagent was substituted with 100 μl of solubilizing agent (DMSO). The absorbance was read at 570 nm. The IC_50_ value was determined by fitting the data with a straight line (linear regression) and using the formula Y = a * X + b.

### Magnetofection

HeLa cells were seeded into 12- well, flat-bottom culture plates and incubated overnight at 37 °C in a 5% CO_2_ incubator. The transfection experiments were carried out when the cells reached 60–70% confluence (25,000–50,000 cells per well). Before transfection, the medium was substituted with 400 *μ*l of fresh DMEM without FBS and antibiotics. Subsequently, 100 μl each of the core-shell was added to the cells, and magnetofection was carried out by placing neodymium magnets under the well plates along with a control, i.e., without the magnetic field for the same duration of 20 minutes. The medium in the plates was drained and added with fresh medium containing 10% FBS and antibiotics and cultured for 48 hrs. Transfection with a commercially available transfection reagent, Lipofectamine 2000, was also conducted for up to 45 minutes to compare the efficiency of the core shell.

### Cellular uptake in HeLa cells

In order to evaluate the capacity of intracellular uptake by Magnetofection, the core shell was complexed with the Green fluorescent plasmid DNA. The core shell was added to HeLa cells in FBS antibiotic-free medium. The cells were then washed with PBS and trypsinized, then resuspended in PBS and analysed by Flow cytometry. The percentage of transfected cells was assessed using dot plot analysis.

### Statistical analysis

All experiments were performed in triplicate, and results are represented as means with standard deviation. Statistical analyses were performed by one-way analysis of variance (ANOVA) followed by a student’s t-test.

## Results

Magnetic Iron oxide nanoparticles were synthesized in order to use as a carrier for molecules to be inserted in the cells. The core shell also was prepared by coating the SPIONs without losing its magnetic property. The properties of the material ie., size, shape, charge, and the integration of the functionalized substance including nucleic acid, were confirmed.

**Fig 1:**
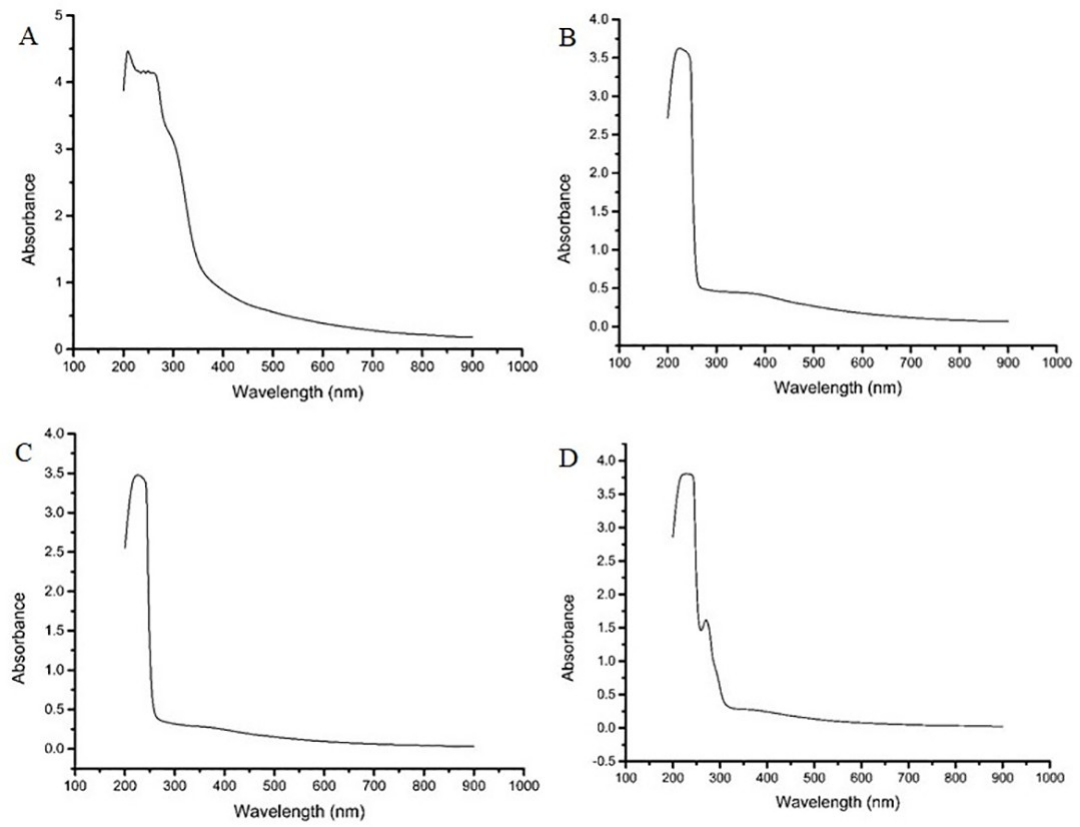
UV-Visible spectra of four phases of core-shell preparation. (A) Naked nanoparticles (B) Starch coated nanoparticles (C) PEI coated nanoparticles (D) Core-shell.

The formation of SPIONs was confirmed by using UV- Visible spectra. It showed a distinct absorption peak at 210 nm (Fig 1A), which confirms the presence of iron oxide nanoparticles. After functionalization with starch, the peak was concealed entirely (Fig 1B), which confirms that SPIONs are functionalized. After coating PEI over functionalized SPIONs showed a slight decrease in absorbance (Fig 1C) in the same range, which can be ascribed to the gelatinous nature of PEI. After the final coating of DNA onto SPIONs, (Fig 1D), a broad band was observed (240–270 nm) in the UV region and a maximum peak at 258 nm confirming the presence of DNA [14].

**Fig 2:**
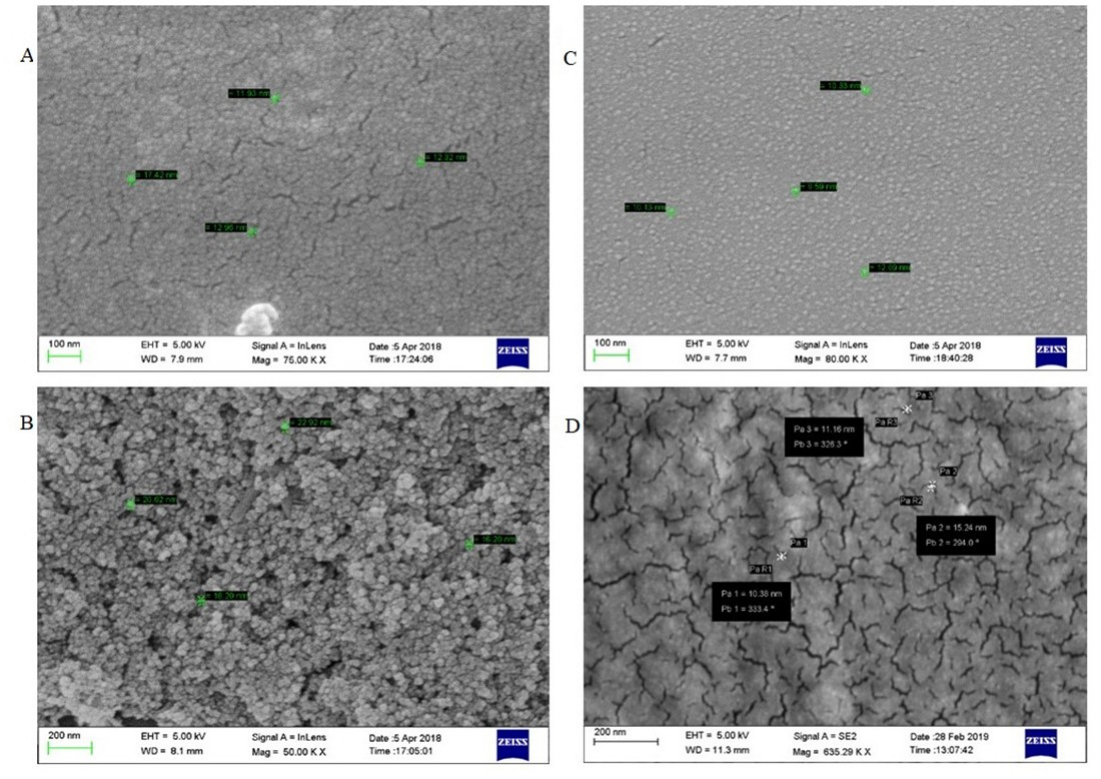
SEM images of four phases of core-shell preparation. (A) Naked nanoparticles (B) Starch coated nanoparticles (C) PEI coated nanoparticles (D) Core-shell.

**Fig 3:**
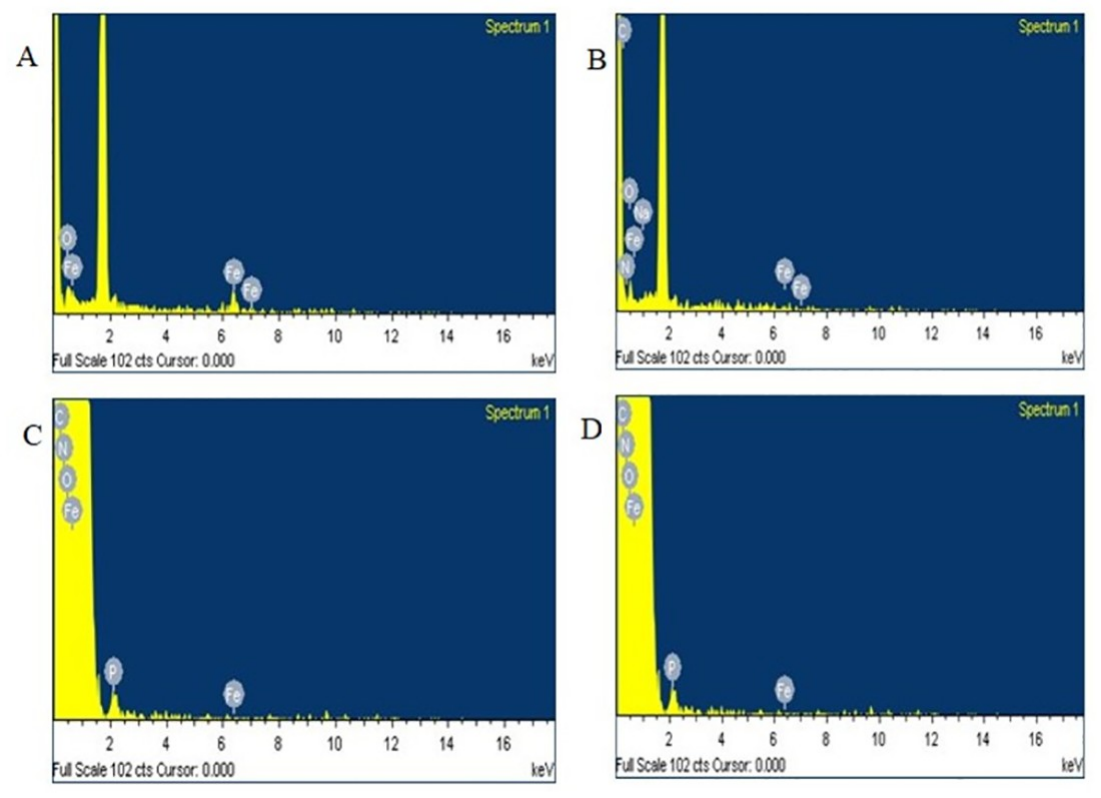
SEM-EDAX images of four phases of core-shell preparation. (A) Naked nanoparticles (B) Starch coated nanoparticles (C) PEI coated nanoparticles (D) Core-shell.

**Fig 4:**
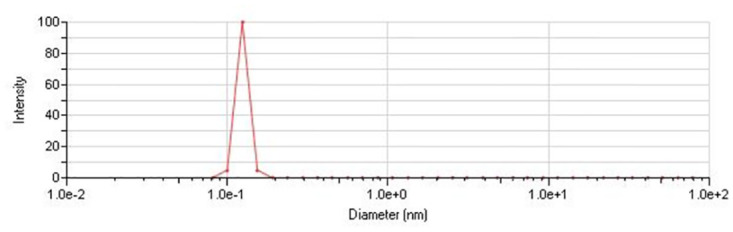
DLS image of core-shell.

Images of the naked and the core shell were taken by Scanning Electron Microscopy (SEM). The naked nanoparticles (Fig 2A) showed uniform spherical particles having a mean diameter of 10 nm, and starch-coated nanoparticles (Fig 2B) are seen with sizes from 10–15 nm. The polymer-coated nanoparticles (Fig 2C) yielded uniform shape and size. The final core shell had an average diameter of 10 nm and a spherical shape (Fig 2D). The spherical-shaped particles are considered less toxic compared to other shapes [15]. The spherical structure of particles facilitates an increased surface area and biocompatibility. The Energy Dispersion Spectrum (Fig 3A) shows the peaks for only Iron (Fe) and oxygen (O). Since there is no indication of other peaks, we can confirm the presence of iron oxide in the nanoparticles. The spectrum for starch (Fig 3B) shows the peaks for C and N. Whereas the spectrum for polymer (Fig 3C) used for coating has peaks for C, N, and O. Similar peaks can be seen in the spectrum for core shell also with an additional peak for P (Fig 3D). DLS imaging also confirm the above-mentioned size of the core-shell (Fig 4).

**Fig 5:**
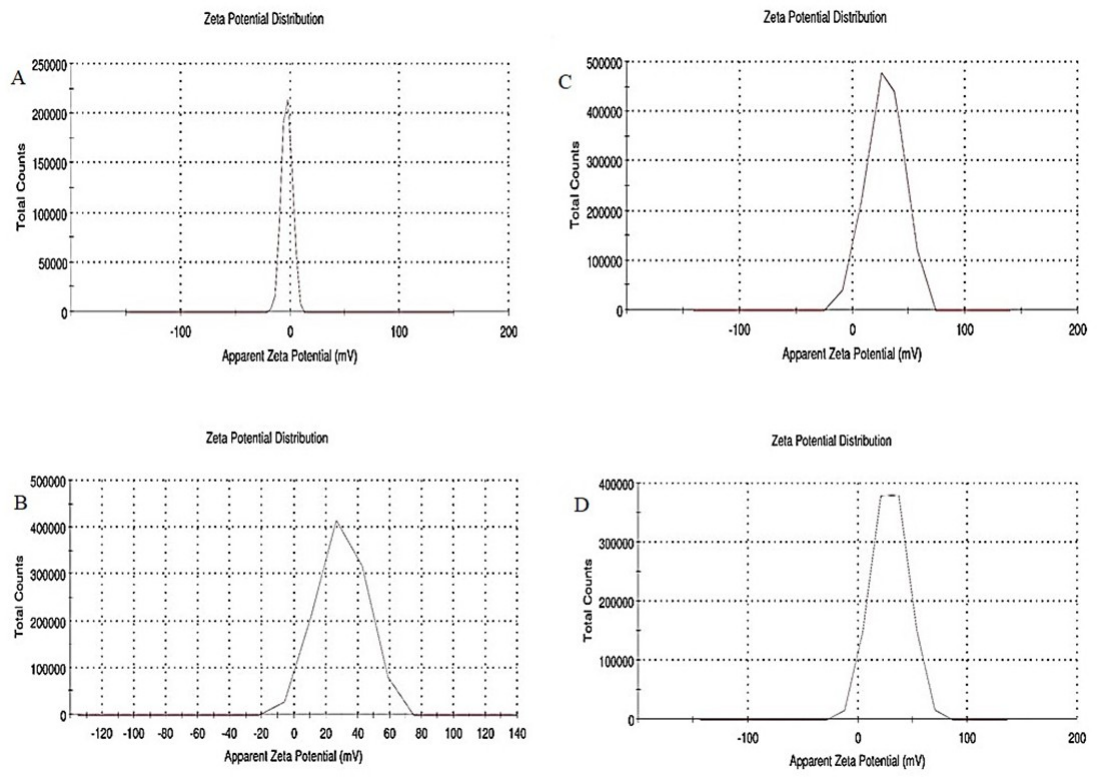
Zeta potential graph of four phases of core-shell preparation. (A) Naked nanoparticles (B) Starch coated nanoparticles (C) PEI coated nanoparticles (D) Core-shell.

The Zeta potential measurement of naked nanoparticles (Fig 5A) was found to be -2.93 mV. The value is+- negative and low as they have a negative charge, and the particles agglomerate, respectively, indicating low stability before functionalizing. The potential of starch-coated nanoparticles is 29.8 mV (Fig 5B), and the polymer-coated nanoparticles have a value of 33.2 mV (Fig 5C), indicating the successful coating of the polymer onto the nanoparticles. The DNA coated has a slightly decreased value since they possess a negative charge on the surface and are found to be 29.1 mV (Fig 5D).

**Fig 6:**
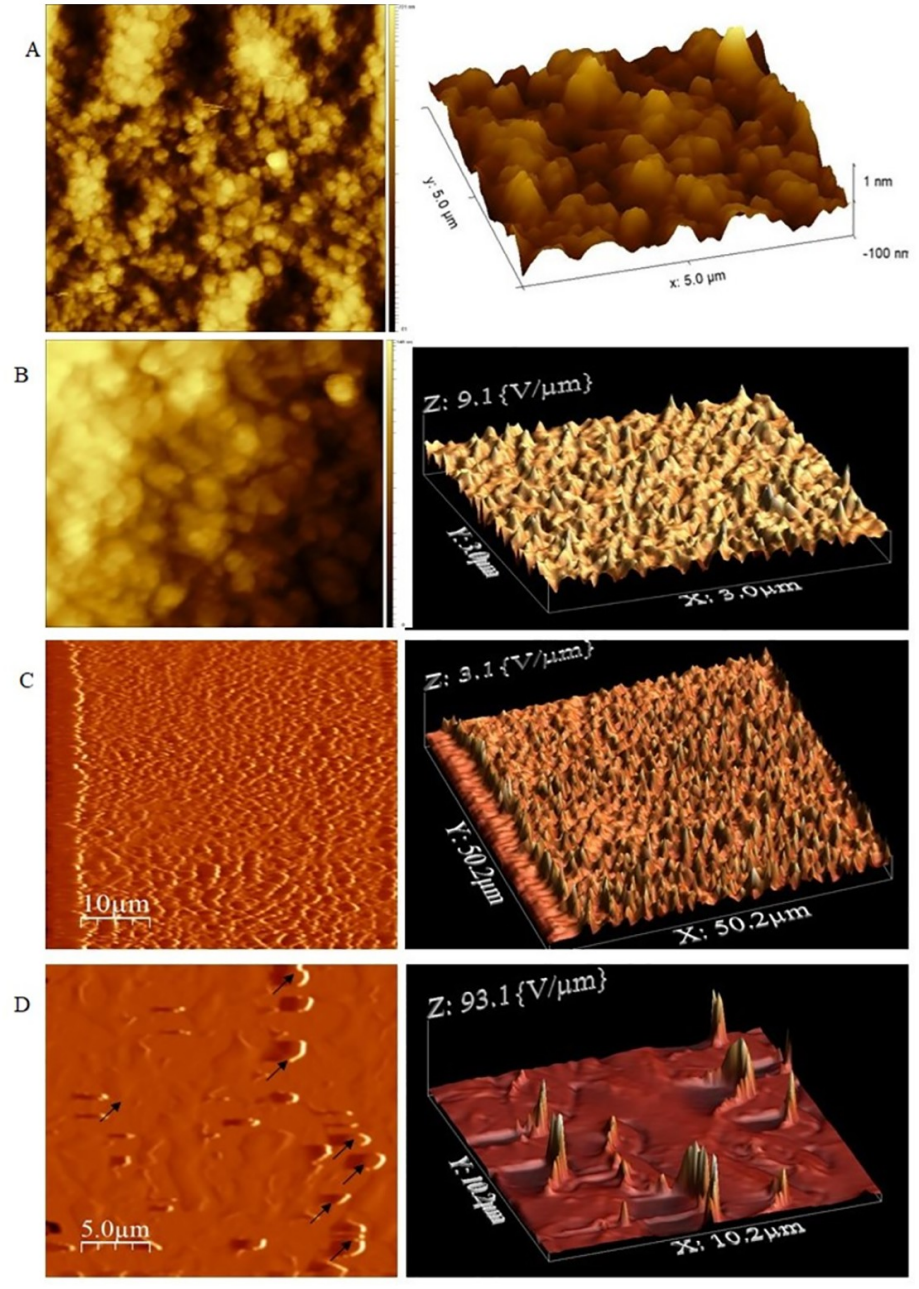
AFM images of four phases of core-shell preparation. (A) Naked nanoparticles (B) Starch coated nanoparticles (C) PEI coated nanoparticles (D) Core-shell.

The AFM images of naked nanoparticles (Fig 6A) also shows that the particle has a spherical surface. The 2D and 3D images show clear spherical nanoparticles with uniform shapes. In the 2D image for starch coating, a layer coated onto the surface can be seen, and 3D images show the coating of the starch is uniform (Fig 6B). The polymer coating of nanoparticles is shown in Fig 6(C). The 2D and 3D images show the clear coating and the uniform shape, respectively. The final coating of DNA onto nanoparticles is seen in Fig 6(D). In the 2D image, we can see the DNA as a white layer on the nanoparticles [16], but in the 3D image, it is seen as peaks. It also confirms that the DNA has bound to the polymer-coated nanoparticles.

**Fig 7:**
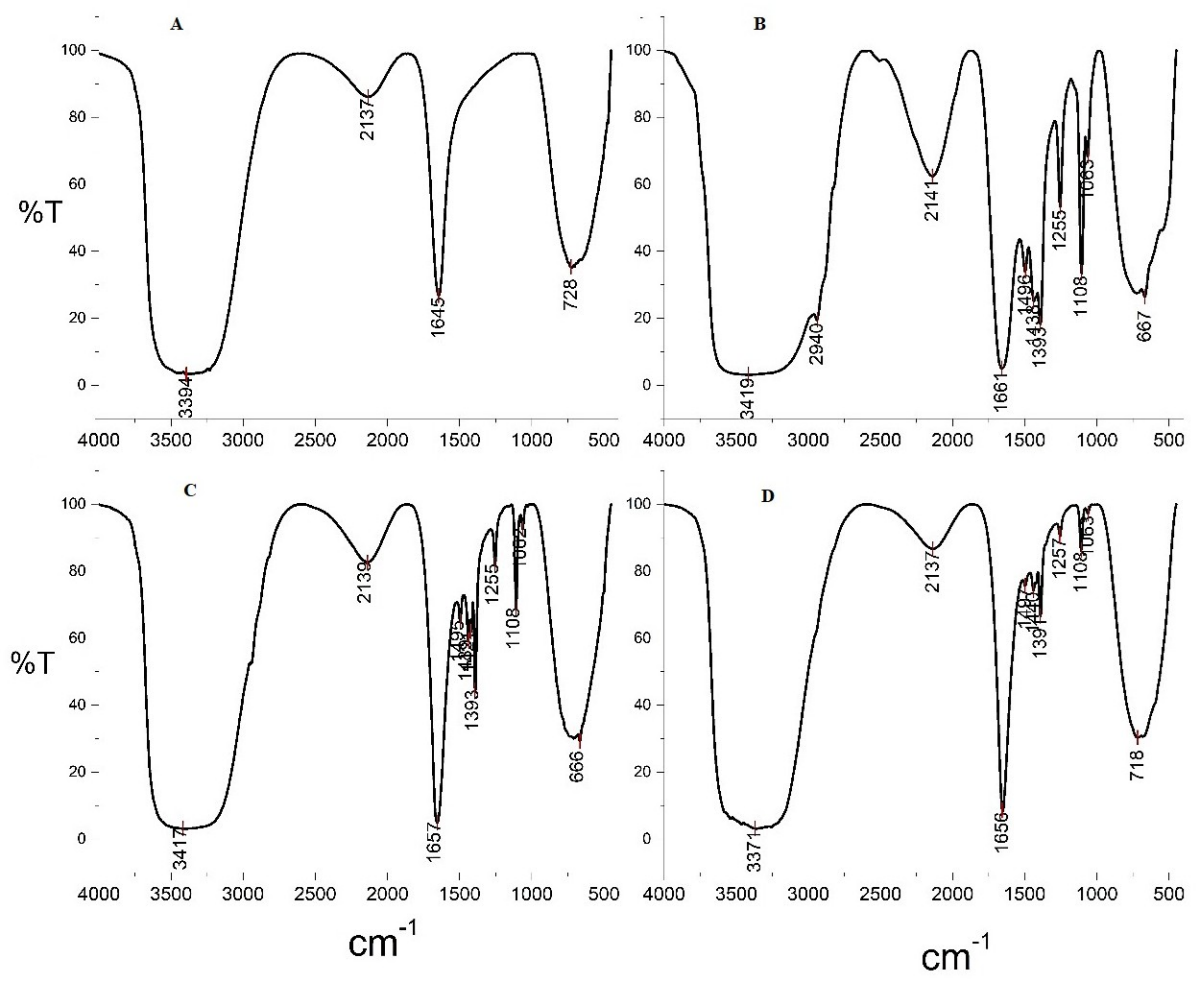
FT-IR spectra of four phases of core-shell preparation. (A) Naked nanoparticles (B) Starch coated nanoparticles (C) PEI coated nanoparticles (D) Core-shell.

The FTIR spectrum of naked nanoparticles (Fig 7A) shows a peak at 728 cm^-1^ due to the presence of iron-oxygen (FeO), which confirms that the synthesized particles are iron oxide [17]. The peaks at 1645 cm^-1^ and 3394 cm^-1^ are due to the vibration of absorbed water molecules and the O-H stretching vibration of a surface hydroxyl group. The peak at 2137 cm^-1^ is due to the C-H stretching vibration of the methyl group present in N, N dimethylformamide [17, 18]. Fig 7(B) shows two characteristic peaks that appeared between 1000 cm^-1^ and 1200 cm^-1^, attributed to the C-O bond stretching of the starch. The bands in 1065 and 1108 cm^-1^ were associated with the acetal group of amylose and amylopectin of starch. The peak observed at 1063 cm^-1^ relates to the C-C and C-O stretching of the polysaccharide backbone. Fig 7(C) for PEI shows peaks at 1657 cm^-1^ (C = O amide), 1393–1657 cm^-1^ (NH_2_- scissoring vibration and C-H stretching vibration) [19, 20]. Therefore, we confirm that the PEI has been coated. The cationic side chains yield a starch-graft-PEI copolymerization [21, 22]. The structure of PEI and DNA has common NH groups, so there are not many changes in the peaks. The peak at 1656 cm^-1^ is characteristic of C = O and C = N from the DNA (Fig 7D). The base sugar vibrations are seen at 1440 and 1497 cm^-1^. The slight shift in the absorption peak at 1063 cm^-1^ and 1257 cm^-1^ correspond to asymmetric phosphate stretch [23, 24].

**Fig 8:**
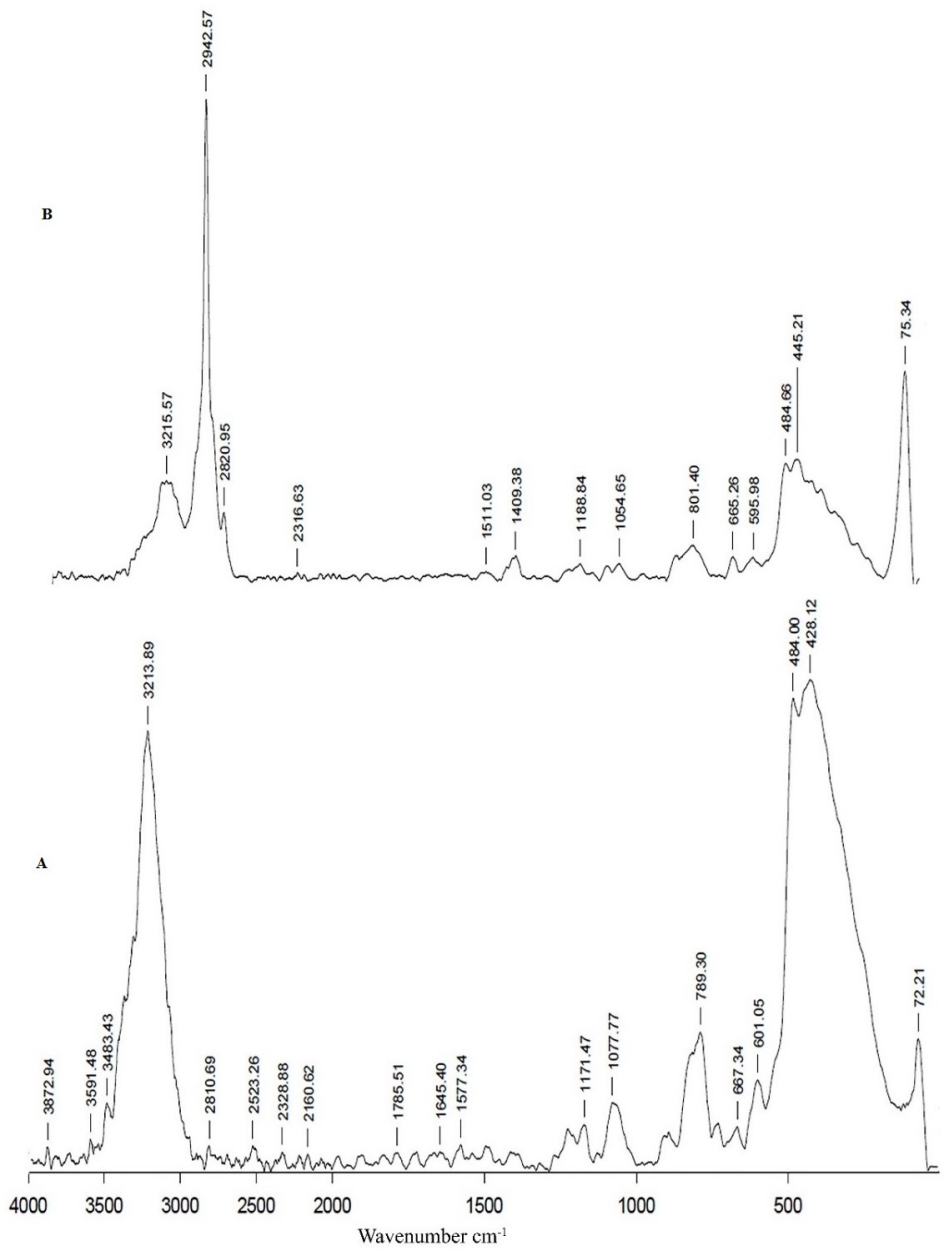
Raman spectra of four phases of core-shell preparation. (A)Naked nanoparticles (B) Starch coated nanoparticles.

**Fig 9:**
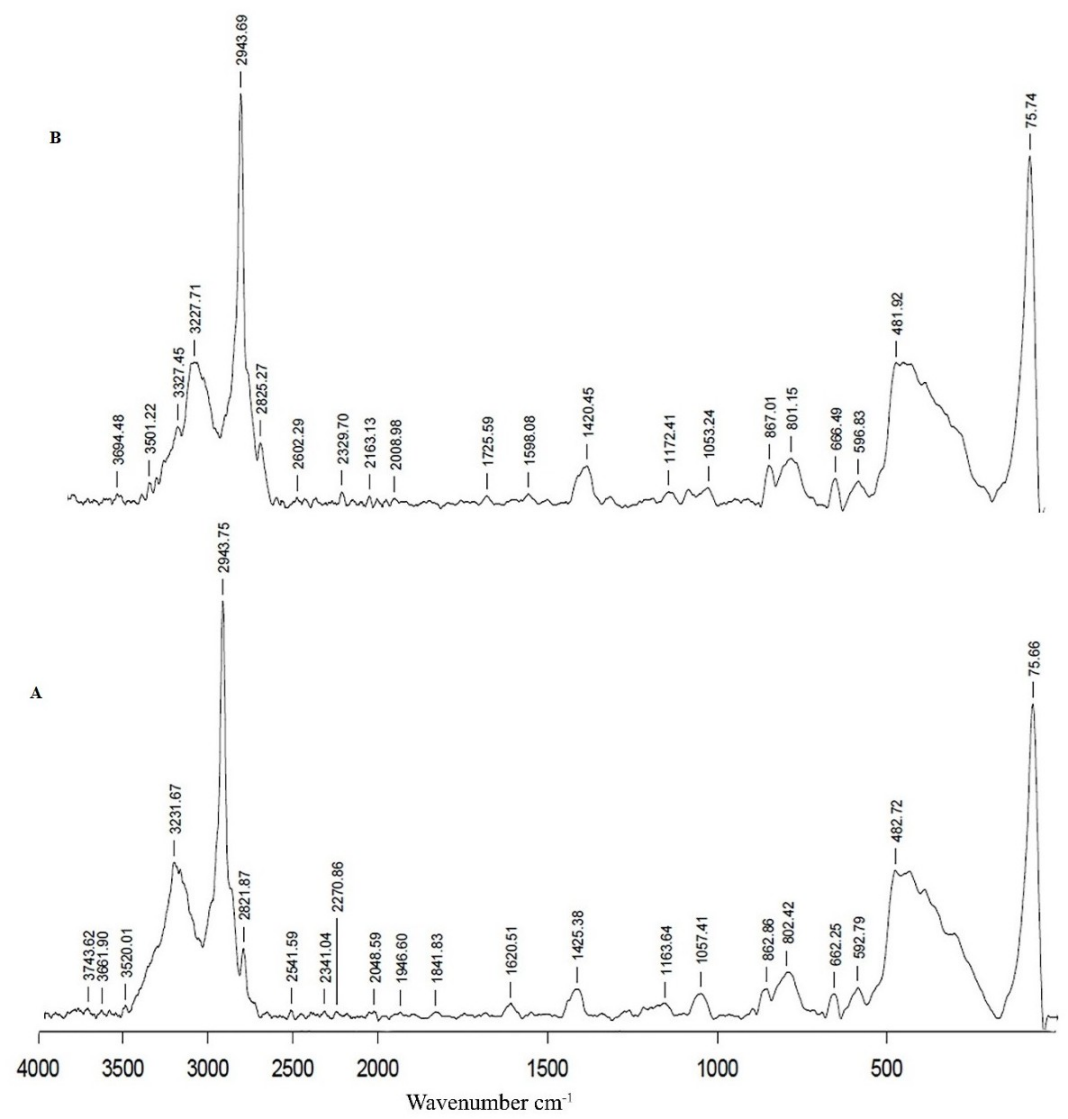
Raman spectra of four phases of core-shell preparation. (A) PEI coated nanoparticles (B) Core-shell.

The Raman spectra for naked nanoparticles (Fig 8A) show the fingerprint region from 450–650 cm^-1^, which is assigned to FeO. Two other peaks, 428 and 484 cm-^1^, are also assigned to FeO in the spectrum [25, 26]. The spectrum for starch-coated nanoparticles (Fig 8B) shows bands at 1188 cm^-1^ that can be attributed to the CH_2_OH-related deformation of amylose [27]. The band in the region 1409 cm^-1^ indorsed to CH_2_ deformation due to the bending mode of C-H. The decrease in the intensity of the bands of iron oxide confirms the functionalization of starch over iron oxide. PEI-coated particles (Fig 9A) show a band at 1425 cm^-1^ attributed to CH_2_ bending. The weak bands at 802 cm^-1^ and 882 cm^-1^ may be due to the rocking vibrations of ethylene groups. The band at 1620 cm^-1^ is due to the amine bending of NH_2_. Amino groups on the core shell might be represented with the peak observed at 1598 cm^-1^ [28] (Fig 9B).

**Fig 10:**
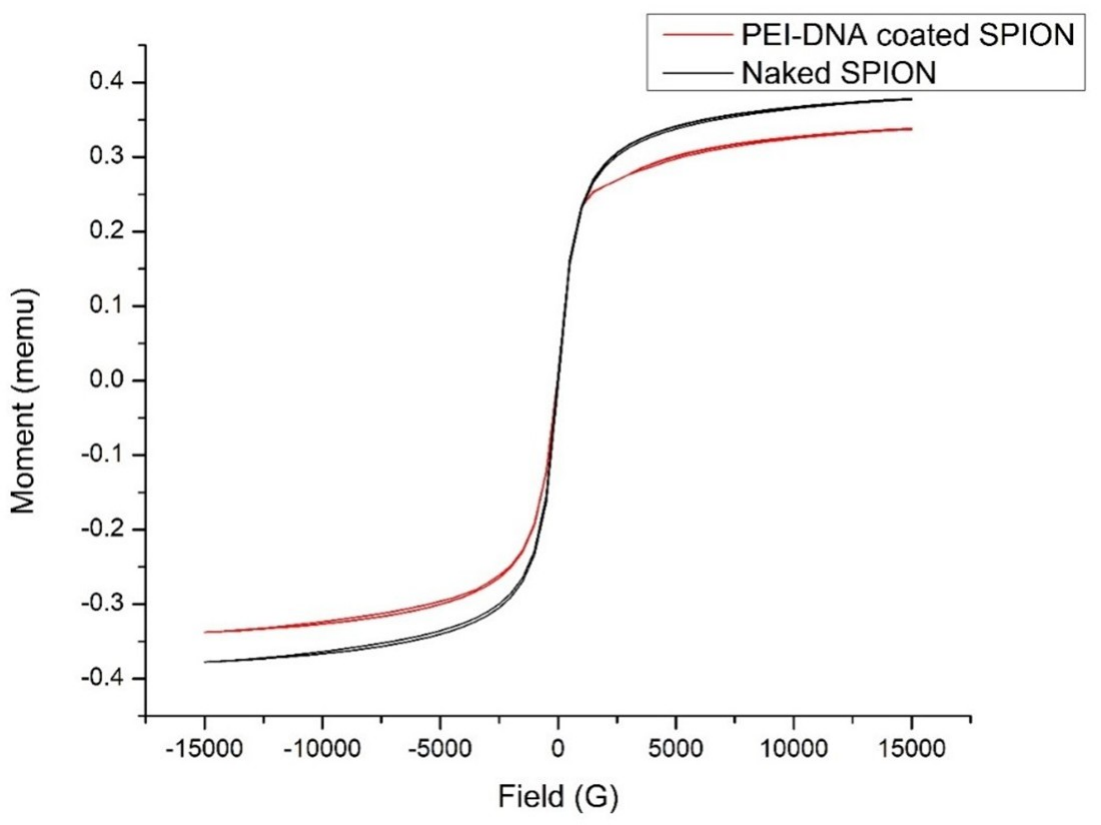
VSM spectra of naked SPIONs and PEI-DNA coated SPIONs.

The magnetic property of the naked nanoparticle and the coated nanoparticles were measured by a Vibrating sample magnetometer. The hysteresis loop of nanoparticles confirms that the nanoparticles were superparamagnetic at room temperature with no loss in hysteresis (Fig 10). The saturation magnetization M_s_ at 300 K was 37.82 emu/g, and that of coated is 33.82 emu/g. The reduction in the value is due to the coating of nanoparticles with a non-magnetic polymer. Based on the calculation, the magnetic particle size (Dm) in the sample was found to be 4.48 nm. There is a size difference compared with SEM, possibly due to a magnetically "dead layer" present on the surface [29].

**Fig 11:**
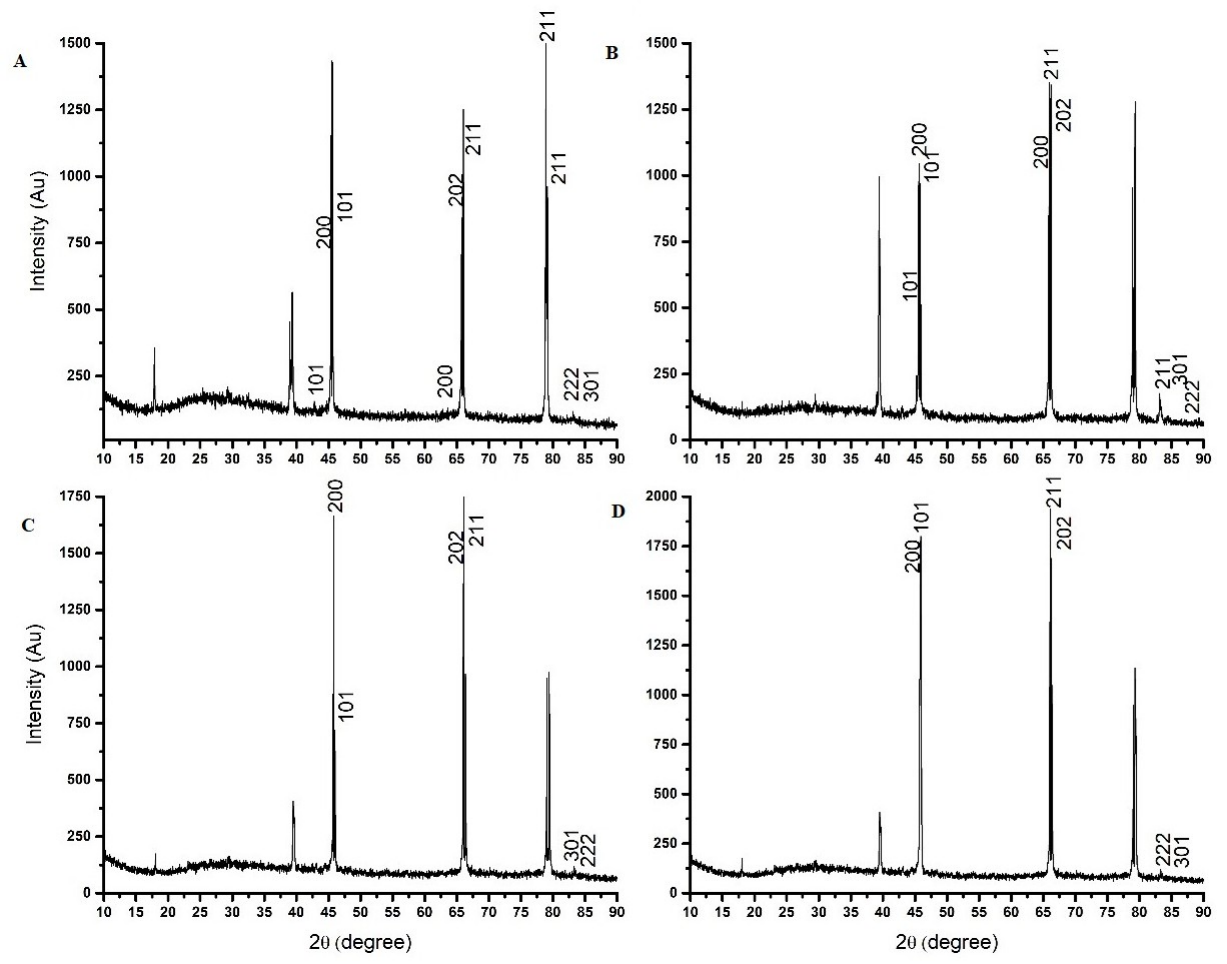
XRD spectra of four phases of core-shell preparation. (A) Naked nanoparticles (B) Starch coated nanoparticles (C) PEI coated nanoparticles (D) Core-shell.

The crystalline phase of the iron oxide nanoparticle was identified at different stages of the preparation of the core shell by XRD. The Fig 11(A) shows the XRD pattern of naked nanoparticles. The characteristic peaks for iron oxide (FeO) were detected 2*θ* = 43.16, 45.46, 45.58, 62.36, 66.25, 66.45, 79.93, 84.03, and 84.28. The particles were found to have a cubic crystalline structure. The XRD pattern for starch coated is shown in Fig 11(B) and the 2*θ* = 45.15, 45.61, 45.73, 65.77, 69.66, 83.36, 84.34, and 84.69. There is a slight change in the peak, which may be due to the starch coating. The XRD for PEI-coated nanoparticles is shown in Fig 11(C) 2*θ* values are 39.79, 39.90, 45.58, 45.70, 46.28, 66.18, and 84.69. The DNA-coated nanoparticles are shown in Fig 11(d) and the 2*θ* are 45.68, 45.80, 66.26, 84.49, and 84.79. The shift in peaks is due to the coating of polymer and DNA over the molecule. The presence of strong amine group might cause the shift. The Miller indices for the entire spectrum were detected at 101, 200, 202, 211, 222, and 301. Iron oxide (FeO) was found to be present at all stages of the core shell. The reflecting peak at 2*θ* = 45.46 was chosen for calculation. The estimated average diameter of iron oxide particles was 15.41 nm. The XRD analysis shows that iron oxide (FeO) was present at all the synthesis stages.

**Fig 12:**
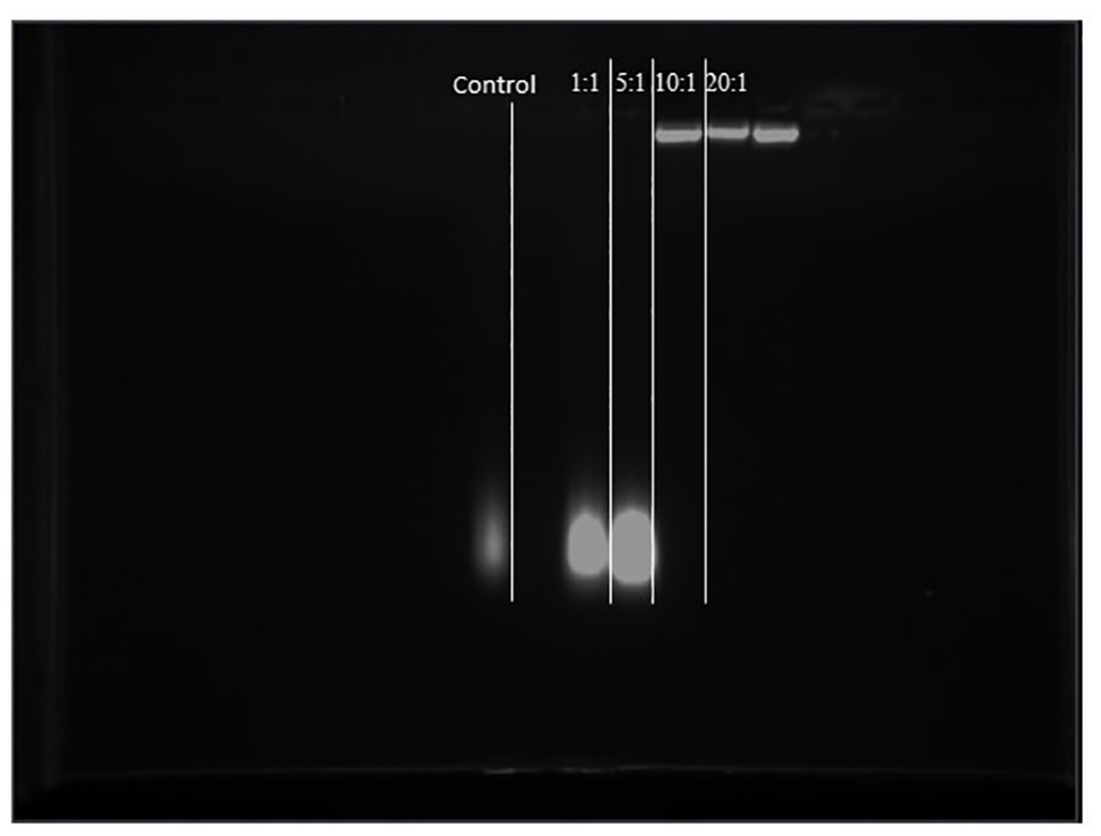
Gel retardation assay of PEI/DNA complexes at different nanoparticles ratios.

The binding affinity of the core-shell for pDNA was evaluated by agarose gel (1.0% w/v) retardation assay (Fig 12). The cores-shell was able to retard the pDNA when used at a mass ratio of core shell to pDNA = 10 or above, indicating the successful DNA binding via electrostatic interaction.

**Fig 13:**
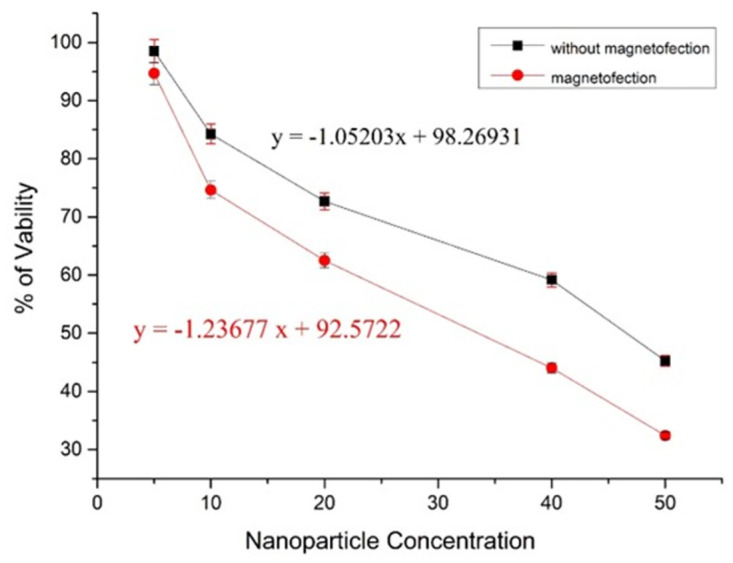
Cell cytotoxicity assay graph showing viability at different concentrations of nanoparticles (µg/ml).

The MTT assay results showed that the cell viability decreased by increasing core-shell concentration. The IC50 value for magnetofection was found to be 34.4 μg, and transfection without magnetic field exposure was 46.2 μg (Fig 13). The value is critical for choosing the right concentration for gene delivery.

**Fig 14:**
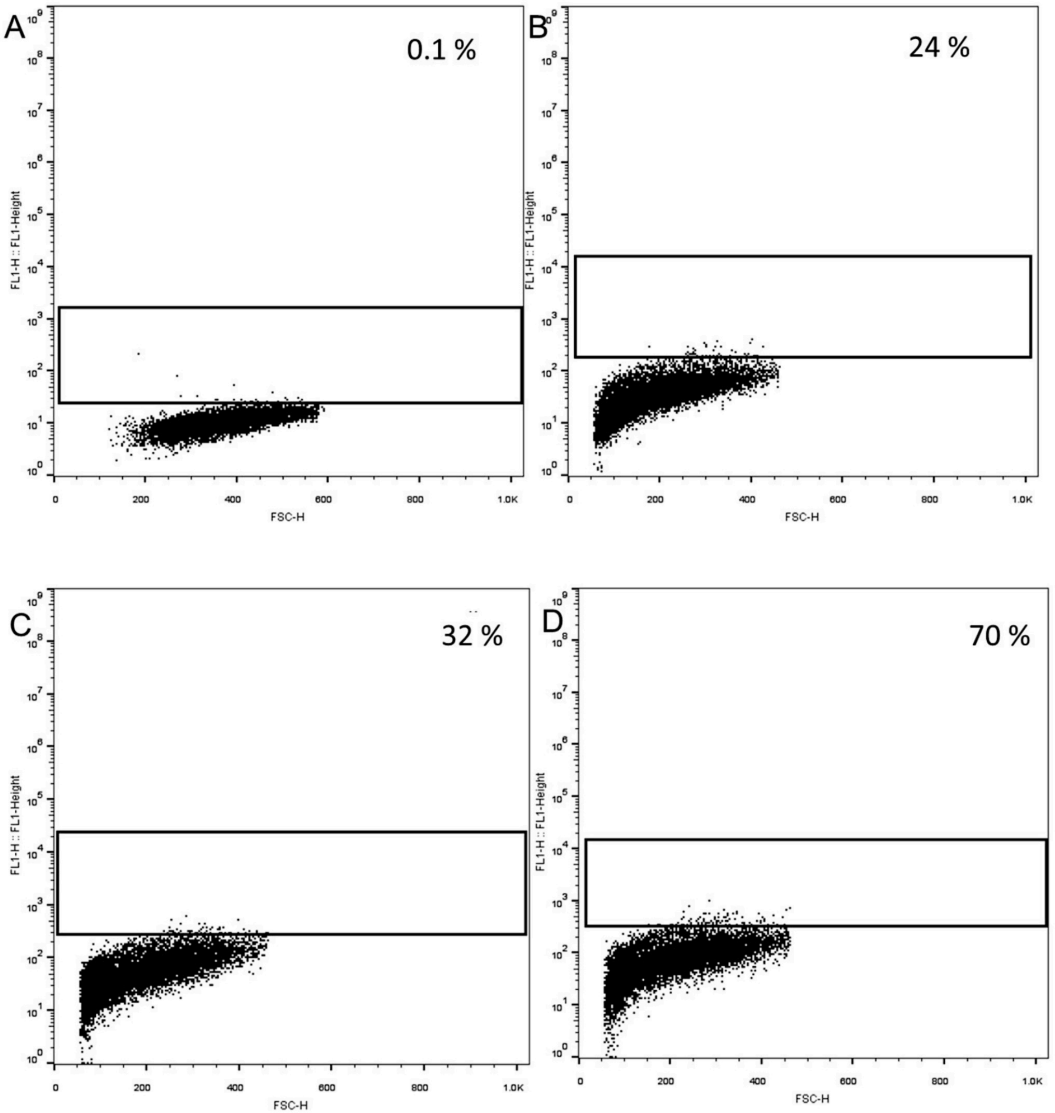
Flow cytometry analysis after transfection with GFP Plasmid. (A) Control (B) without magnetofection (C) Lipofectamine 2000 (D) Magnetofection.

**Fig 15:**
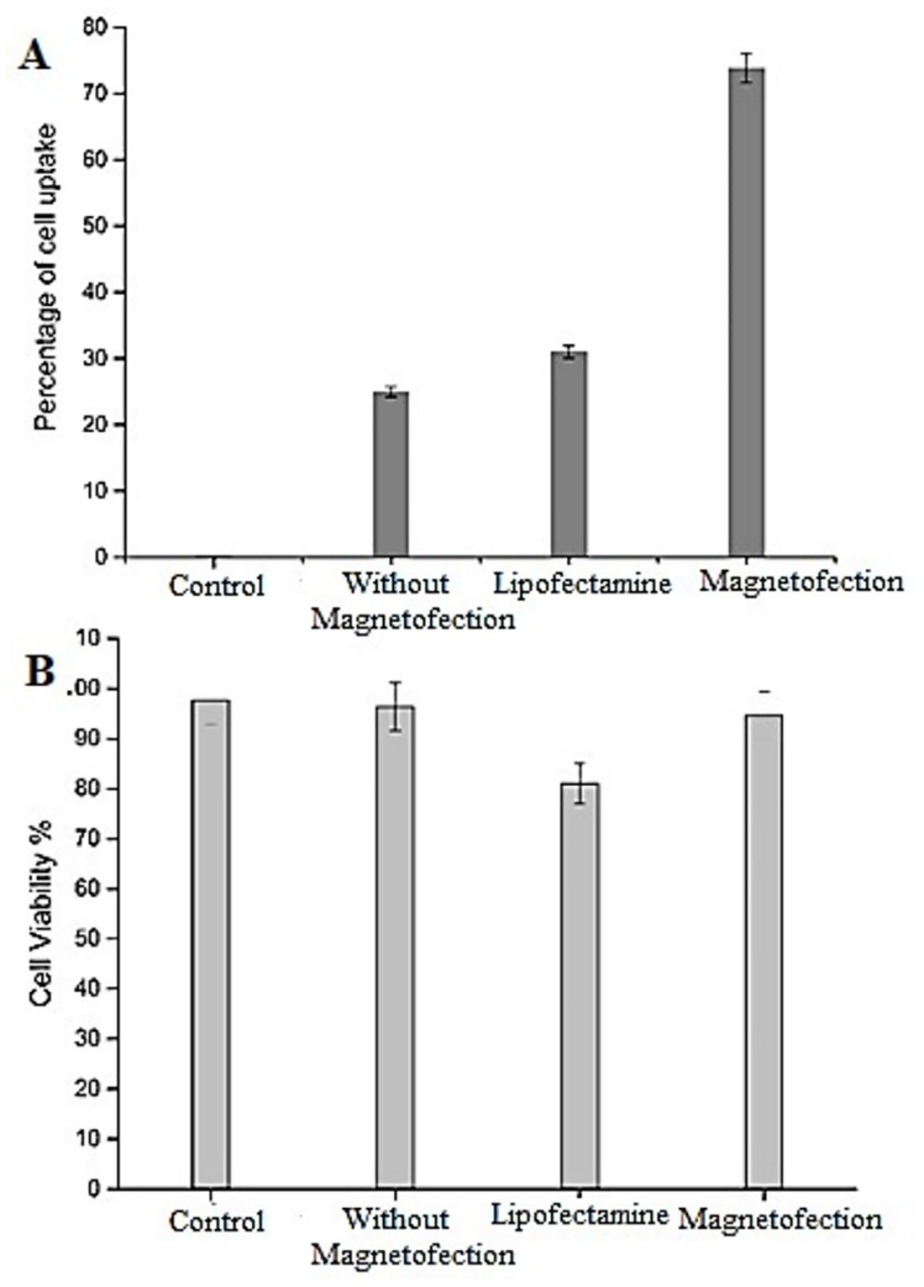
Gene transfection efficiency. (A) quantification of GFP- cellular uptake by flow cytometry analysis after transfection, (B) Cell viability after transfection in HeLa cells.

Flow cytometry evaluated the uptake capacity and efficiency of the core shell after magnetofection in *HeLa* cells by the complexation of GFP DNA with the core shell. The green fluorescent signal from the GFP was used to evaluate the uptake capacity. The flow cytometry results are represented as a dot plot and gated to analyse the transfection. The rectangle box represents the gated area, and the percentage of transfected cells is shown in the upper-right corner of the graph (Fig 14). The flow cytometry analysis showed that the magnetofected core shell had 70% of uptake capacity (Fig 14D) and transfection without magnetofection had 24% of uptake capacity (Fig 14B). Transfection with Lipofectamine 2000 had an uptake of 32% (Fig 15A). Transfection efficiency of core-shell via magnetofection had higher cellular uptake with 40% efficiency when compared to Lipofectamine 2000. The viability of cells after magnetofection with core-shell and transfection with Lipofectamine 2000 was 95% and 80%, respectively (Fig 15B). Though the Lipofectamine had 32% of cellular uptake, it decreased the viability. The core-shell magnetofection has high efficiency in less duration when compared to Lipofectamine 2000 mediated transfection.

## Discussion

A perfect carrier based delivery system especially using magnetic nanoparticles is convenient in medical applications. The specific gene, choice of target, and limitation of delivery systems are to be considered when synthesizing nanoparticles. Size, toxicity, binding capacity, and magnetic property of the nanoparticle are essential for successful gene delivery. In our studies, we synthesized superparamagnetic iron oxide nanoparticles using a simple co-precipitation method.

The mean size of the synthesized nanoparticle was 10 nm, which would be highly efficient. The smaller core shell complexes are essential for their successful cellular internalization and gene delivery. Earlier reports suggest that nanoparticles of size 40 nm are efficient for gene delivery [30]. The colloidal stability of nanoparticles with the imparted charge is predicted with the magnitude of the zeta potential, and our particles have a potential of 29 mV. These particles can effectively be used in delivery systems as suggested in the earlier report that the zeta potential of 22 mV is attractive for delivery applications [31].

For effective gene delivery, better binding of the gene and a good core shell is essential. The mass ratio impacts DNA retardation and is substantial above the ratio of five [32]. In the present study, the nanoparticles had a better retardation capacity when compared to previous reports, as the core shell could successfully bind with pDNA via electrostatic interactions and retard pDNA at a mass ratio of 10 or above. A similar study on gel retardation assay of nanoparticles reported that when the concentration of nanoparticles to polymer was more significant than 4, they had a strong binding [33]. Binding capacity increases gene transfection efficiency.

The toxicity of nanoparticles is one of the critical factors for delivery applications. The toxicity of the iron oxide nanoparticles in earlier reports was above 100 *μ*g/ml and 50 *μ*g/ml, respectively [34, 35]. Our nano core shell was found to be nontoxic at a concentration of up to 200 *μ*g/ml, which is five times greater than the concentration at which the core shell showed optimum transfection efficiency. Lower toxicity confirms an efficient synthesis of nanoparticles in the present study.

Cellular uptake is a significant factor for efficient gene transfer. The process is affected by the mode of transfection and duration. The efficiency of iron oxide nanoparticle-mediated gene transfection was compared with Lipofectamine 2000 in HeLa cells. The transfection efficiency of the core shell was almost 40% higher than Lipofectamine 2000. Reports on magnetofection reveal that the cellular uptake of 45% and 40%, respectively, in similar experiments using iron oxide nanoparticles wherein a specially designed instrument with a rotating table fitted with neodymium magnets was used for magnetofection, which provided a uniform magnetic field [36, 37]. The core shell prepared in the present study has an efficiency of 70% cellular uptake with a magnetofection duration of 20 minutes against the earlier report of 1 hour [36]. The cell viability during the transfection is also to be considered, as the Lipofectamine negatively affects the survival of cells [38]. Transfection efficiency reported in gold nanoparticles was up to 50%, and the iron oxide nanoparticles up to 30% [7, 39]. The results also confirm that our nanoparticle had higher uptake efficiency than Lipofectamine 2000 [40–42].

In conclusion, in the present study, we could successfully synthesize stable mean sizes up to 10 nm iron oxide nanoparticles and simple, efficient gene delivery by magnetofection. These nanoparticles had good size, better magnetic properties, biocompatibility, low toxicity, and higher transfection efficiency. This method would be efficient for any gene delivery studies.
